# Several Polymorphisms of *KCNQ1* Gene Are Associated with Plasma Lipid Levels in General Chinese Populations

**DOI:** 10.1371/journal.pone.0034229

**Published:** 2012-03-29

**Authors:** Xing-dong Chen, Ya-jun Yang, Shu-yuan Li, Qian-qian Peng, Li-juan Zheng, Li Jin, Xiao-feng Wang

**Affiliations:** 1 State Key Laboratory of Genetic Engineering and MOE Key Laboratory of Contemporary Anthropology, School of Life Sciences and Institutes of Biomedical Sciences, Fudan University, Shanghai, China; 2 China Medical City Institute of Health Sciences, Taizhou, Jiangsu, China; Universidad Europea de Madrid, Spain

## Abstract

**Background:**

Potassium voltage-gated channel, KQT-like subfamily, member 1 (*KCNQ1*) is thought to be an important candidate gene of diabetes. Several single nucleotide polymorphisms (SNPs) in a 40-kb linkage disequilibrium (LD) block in its intron 15 have been identified to be associated with diabetes in East Asian populations in recent genome-wide association studies. The aim of this study was to investigate whether *KCNQ1* polymorphisms influence the levels of the metabolic phenotypes in general Chinese populations.

**Methodology/Principal Findings:**

We investigated the associations of two SNPs (rs2237892 and rs2237895) in the aforementioned 40-kb LD block, a missense variant rs12720449 (P448R) in exon 10, and a synonymous variant rs1057128 (S546S) in exon 13 with metabolic phenotypes in a Uyghur population (n = 478) and replicated these associations in a Han population (n = 2,485). We found that rs2237892-T allele was significantly associated with decreased triglyceride levels (*p_combined_* = 0.001). The minor G allele of the rs12720449, with sharp difference of the allelic frequency between European and East Asian populations (0.2% versus 14%, respectively), was associated with a lower triglyceride levels than G allele in Uyghur subjects (*p* = 0.004), in Han subjects (*p* = 0.052), and in subjects of meta-analysis (*p_combined_* = 0.001). Moreover, the minor A allele of the rs1057128 was also associated with decreased triglyceride levels in meta-analysis (*p_combined_* = 0.010).

**Conclusions:**

To the best of our knowledge, this is the first report associating a missense mutation of *KCNQ1*, rs12720449, with triglyceride levels. Rs2237892, representing the 40-kb LD block, is also associated with triglyceride levels in Han population. Further studies are required to replicate these findings in other East Asian populations.

## Introduction

The potassium voltage-gated channel, KQT-like subfamily, member 1 (*KCNQ1*), which encodes the pore-forming voltage-gated K^+^ channel subunit KvLQT1 and is widely expressed in myocardial tissue, plays a key role in the repolarization of the cardiac action potential and in the transport of water and salt in epithelial tissues [1]–[3]. Loss-of-function and gain-of-function mutations in this gene have been associated with cardiac long QT syndrome, Lange-Nielsen cardioauditory syndrome, atrial fibrillation, and congenital deafness [2]–[6].

*KCNQ1* is expressed in the inner ear, stomach, intestine, liver, kidney, and pancreatic islets [5]. In the pancreas, *KCNQ1* contributes to the regulation of insulin secretion, and a blockade of the KvLQT1 channel may lead to increased insulin secretion [7], [8]. Recently, two independent genome-wide association studies (GWAS) revealed that several SNPs (including rs2237892, rs2237895, rs2283228, and rs2237897) within a 40-kb linkage disequilibrium (LD) block in intron 15 of *KCNQ1* were consistently associated with type 2 diabetes and the impairment of insulin secretion in East Asian populations [9], [10]. For these SNPs, the associations with type 2 diabetes have been replicated, predominantly not only in East Asian ethnic groups, including Chinese [11], [12] and Singaporean populations [13], but also in Euro-Caucasian populations from Denmark [10], [14] and Sweden [15]. Moreover, rs2237892 and rs2237895 have also been associated with metabolic phenotypes, such as glucose and body mass index (BMI) levels, in East Asian populations [13], [16].

Because *KCNQ1* is critical for the regulation of insulin secretion [5] and insulin is important for the regulation of metabolic phenotypes, we hypothesized that genetic polymorphisms in the *KCNQ1* gene may underlie differences in metabolic phenotypes, such as triglyceride (TG) and total cholesterol (TC). In addition to SNPs located in the 40-kb LD region, the pathogenesis effects of another two important SNPs in the exon regions of the *KCNQ1* gene, rs1057128 and rs12720449, need to be explored. Synonymous variant rs1057128 (S546S), located in exon 13 of the *KCNQ1* gene, has been found to be associated with various cardiac arrhythmias, such as Long-QT syndrome, atrial flutter, and atrial fibrillation, in European and Chinese individuals [17], [18]. Non-synonymous variant rs12720449 (P448R), located in exon 10 of *KCNQ1*, was also found to be associated with Long-QT syndrome [19], [20]. The minor allele frequency (MAF) of this SNP is distinct between Europeans and East Asians (0.2% and 5.8%, respectively), according to the NCBI SNP databank (http://www.ncbi.nlm.nih.gov/projects/SNP/snp_ref.cgi?rs=12720449). No prior studies have investigated the association between these two SNPs (rs1057128, rs12720449) and metabolic phenotypes.

The aim of the present study was to investigate the effects of rs1057128, rs12720449, and two SNPs (rs2237892 and rs2237895) within the aforementioned 40-kb LD block on several metabolic phenotypes. We first observed the associations of these four SNPs with BMI, waist-to-hip ratio (WHR), systolic blood pressure (SBP), diastolic blood pressure (DBP), glucose (Glu), triglyceride (TG), and total cholesterol (TC) in a Uyghur population, an ethnic group with European and East Asian ancestry. Then we observed the associations in a Han population, the ethnic group that accounts for 94% of the Chinese populations, with a larger sample size.

## Results

### The genotypic and allelic frequencies of the studied SNPs

The genomic characteristics of rs1057128, rs2237892, rs2237895, and rs12720449 were summarized in Table 1. All SNPs were consistent with Hardy–Weinberg expectations in both populations (*p*>0.01). The MAFs of rs12720449 were different for the Han and Uyghur populations in the present study (0.14 and 0.05, respectively). Pair-wise linkage analysis with the four SNPs showed weak LDs (data not shown). To the best of our knowledge, this is the first study investigating SNPs in *KCNQ1* in a Uyghur population. Because the sample size for the Uyghur population was relatively small, we compared the metabolic phenotypes of rare allele carriers (CG/GG for rs12720449) with those of non-carriers in the subsequent analysis. The analysis was performed as previously described in the original GWAS [21].

**Table 1:** Genomic characteristics of the four SNPs in studied subjects.

SNPs	Gene region	Major/Minor Allele	Population	Genotyped frequence	MAF	*p* value*
rs1057128	Exon 13	G/A	Han	1207/1053/211	0.30	0.381
			Uyghur	252/181/34	0.27	0.848
rs2237892	Intron 15	C/T	Han	1161/1048/254	0.32	0.967
			Uyghur	310/130/16	0.18	0.605
rs12720449	Exon 10	C/G	Han	1857/559/60	0.14	0.022
			Uyghur	425/46/2	0.05	0.533
rs2237895	Intron 15	A/C	Han	1186/1048/238	0.31	0.769
			Uyghur	173/222/77	0.40	0.684

* Deviation from Hardy–Weinberg expectation for the variants was tested using the chi-square statistic.

### The association between the four SNPs in KCNQ1 and plasma TG levels

We presented the association results between the four SNPs in *KCNQ1* and plasma TG levels in Table 2. For SNP rs12720449, significantly lower TG levels were observed in CG/GG carriers (0.94±0.35) than in CC carriers (1.08±0.35) (*p* = 0.004) in the Uyghur population. A preliminary significant association was also observed in the Han population, and the mean TG levels of GG, CG, and CC carriers were 0.87±0.30, 1.03±0.92, and 1.08±0.96, respectively (*p* = 0.052). The minor alleles of rs1057128 (A) and rs2237892 (T) were significantly associated with decreased TG levels in Han subjects (*p* = 0.011 for both alleles).

**Table 2:** Association between studied SNPs in KCNQ1 and plasma TG levels.

SNPs	Uyghur	Chinese Han	*p* value		
			Uyghur	Chinese Han	Meta-analysis
**rs1057128**					
GG	1.05±0.33	1.10±1.02			
GA	1.06±0.37	1.02±0.84			
AA	1.15±0.32	1.05±1.05	0.271	**0.011**	**0.010**
**rs2237892**					
CC	1.06±0.34	1.08±0.92			
CT	1.08±0.36	1.07±0.99			
TT	1.00±0.39	0.97±0.73	0.410	**0.011**	**0.001**
**rs12720449**					
CC	1.08±0.35	1.08±0.96			
CG	0.94±0.35	1.03±0.92			
GG		0.87±0.30	**0.004**	0.052	**0.001**
**rs2237895**					
AA	1.05±0.34	1.03±0.91			
AC	1.07±036	1.08±0.94			
CC	1.05±0.34	1.13±1.03	0.851	0.095	0.040

*p* values were inferred from GLM adjusted for age gender smoking and alcohol drinking.

Bold fonts represent significant difference (*p*<0.0125).

To investigate allelic associations with TG levels, we performed a meta-analysis across Uyghur and Han datasets (Table 2). We found that the minor alleles of rs12720449 (G), rs1057128 (A), and rs2237892 (T) decreased TG levels at a multiple correction threshold level of *p*<0.0125 (*p* = 0.001, 0.010 and 0.001, respectively). We also evaluated these associations by including all four SNPs in one model, and the associations between rs1057128, rs2237892, rs12720449 and TG levels remained significant (*p* = 0.007, 0.001, and 0.001, respectively).

### The association between the four SNPs and the principal components of the metabolic phenotypes

To further validate our results, we conducted a principal component analysis in the Han population using nine metabolic-related traits: BMI, WHR, SBP, DBP, Glu, HDL, LDL, TG, and TC. First, we identified two principal components (PC) of these traits to generate canonical variables (PC1 and PC2) (Table 3). Next, we performed a classic single-trait approach to test the associations between SNPs and the canonical variables. As shown in Table 3, PC2 is strongly correlated with TG (with an eigenvalue of 0.98). All four SNPs were significantly associated with PC2 at a threshold level of *p*<0.05 (*p* = 0.017, 0.020, 0.043, and 0.050), but these association did not reach the multiple correction threshold level of *p*<0.0125.

**Table 3:** PCA results of parameters of MS in Chinese Han.

variable	Prin1	Prin2
BMI	0.37	0.05
WHR	−0.34	−0.02
LDL	0.23	−0.06
HDL	0.35	0.10
GLU	0.37	0.06
TG	−0.14	**0.98**
SBP	0.37	0.05
DBP	0.37	0.05
TC	0.37	0.08
Eigenvalue	7.13	0.88
Proportion	0.79	0.10

SNPs	*p* value	*p* value
rs1057128	0.46	**0.017**
rs2237892	0.13	**0.020**
rs12720449	0.18	**0.043**
rs2237895	0.11	**0.050**

The PCA results were gained from Uncorrected Correlation Matrix.

### The association between SNPs in KCNQ1 and other metabolic phenotypes excluding TG

We investigated the relationships of the four SNPs in *KCNQ1* with other metabolic-related parameters, including BMI, WHR, SBP, DBP, Glu, HDL, LDL, and TC. We found a significant association between rs1057128 and SBP in the Han population at the significant level of 0.0125. In the Uyghur population, we found a significant association between rs2237892 and WHR at the significant level of 0.05 (Table 4). We also performed a meta-analysis across the two datasets for the Uyghur and Han populations, but we found that none of associations reached the multiple correction threshold level of *p*<0.0125.

**Table 4:** Association between SNPs in KCNQ1 and other metabolic phenotypes.

	Common genotype	heterozygous	Rare genotype	*p* value
**Uyghur**				
**rs2237892**				
WHR	0.88±0.09	0.88±0.07	0.93±0.20	0.020
**Chinese Han**				
**rs1057128**				
SBP	126.29±18.24	127.88±19.13	124.97±18.71	**0.011**
**rs12720449**				
LDL	1.74±1.04	1.92±1.02	2.26±0.93	0.015
**rs2237895**				
LDL	1.90±1.04	1.73±1.03	1.64±1.01	0.013
TC	4.28±1.60	4.40±1.75	4.58±2.23	0.020
**Meta-analysis**				
**rs1057128**				
SBP	127.90±20.52	128.66±20.41	125.36±18.84	0.018

*p* values were inferred from GLM adjusted for age, gender, smoking, and alcohol drinking.

## Discussion

In this population-based association study, we replicated the association between rs2237892, a SNP in intron 15 of *KCNQ1* gene, and TG levels. We also identified a novel association between rs1057128 (S546S), which is located in exon 13 of the *KCNQ1* gene, and TG levels. Most importantly, we identified a missense mutation, rs12720449 (P448R), located in exon 10 of *KCNQ1*, associated with TG levels.

Intron 15 of the *KCNQ1* gene spans approximately 70 kb on chromosome 11p15.5. An extremely long LD block, covering approximately 40 kb, was observed in a Japanese population, and several SNPs (including rs2237892, rs2237895, rs2283228, and rs2237897) located in this LD block were found to be associated with diabetes in Japanese individuals [9], [10]. However, the LD block in this 40-kb region was weak in other ethnically distinct East Asian groups, such as Singaporean and Han Chinese populations [13], and the associations of these SNPs with diabetes were not consistently replicated in these East Asian populations [11], [13], [16], [22]. Moreover, the associations of the SNPs in this region with other metabolic phenotypes, such as lipid levels, assessed in East Asian populations were inconsistent [23]. In the present study, we did not observe an association between glucose levels and the two SNPs, rs2237892 and rs2237895, within this region. However, we detected associations between rs2237892 and TG levels, rs2237895 and LDL levels, and rs2237895 and TC levels. In the Japanese population, the diabetes causal variant may be in LD with this 40-kb region. In other East Asian ethnic groups, such as Han Chinese or Singaporean populations, different variants affecting lipid metabolic regulation may be in LD with rs2237892 or rs2237895 [13]. Further studies will need to be performed to identify these functional variants.

In the present study, rs1057128 was associated with TG levels in the Han population. This SNP was originally identified in association with various cardiac arrhythmias [17], [18]. Although rs1057128 (S546S) is a synonymous variant, there is evidence indicating that synonymous mutations can affect the thermodynamic stability of mRNA secondary structures or affect splicing through phenomena such as exon skipping; therefore, synonymous mutations may not be neutral in evolution [24]. Additionally, a small domain between residues 589 and 620 in the *KCNQ1* C terminus may function as an assembly domain for *KCNQ1* subunits [25]. Without this domain, *KCNQ1* C termini fail to assemble, and functional potassium channels are not produced. Rs1057128 is located in the *KCNQ1* C terminus close to this domain and may therefore affect the functional protein through this mechanism.

The most important finding of the present study was the association between rs12720449 in *KCNQ1* and TG levels. rs12720449 (P448R), a missense mutation located in exon 10, is a functional variant, and the G allele of rs12720449 can increase channel current by two-fold [19]. The MAFs of this variant are noticeably distinct among different ethnic groups, with ≈15% in East Asians [20], [26], [27], ≈0.2% in Europeans, and ≈6% in Uyghurs, an ethnic group with European and East Asian ancestry. rs12720449 was originally identified as a polymorphism associated with cardiac arrhythmia in people of European descent and was not generally considered to be associated with polygenic diseases, such as dyslipidemia, possibly as a result of its low frequency (0.2%) in European populations [28], [29]. In the present study, this variant was found to be associated with TG levels in both an East Asian population (Han) and an admixed population (Uyghur). However, the underlying mechanism remains unclear, and further studies are needed to elucidate the roles of rs12720449 in evolutionary biology and complex phenotypes.

We also identified several associations between *KCNQ1* polymorphisms and other metabolic phenotypes at a threshold of *p*<0.05 (e.g., rs12720449 and LDL, rs2237892 and WHR, rs2237895 and LDL, and rs2237895 and TC), but we did not detect any association with glucose levels in either Uyghur or Han populations. These variants may result in an increased expression of *KCNQ1* and a subsequent increase in insulin secretion in the pancreatic β cells [8]. Increased insulin inhibits glucose production and stimulates lipid synthesis, which may selectively increase circulating lipid levels without increasing blood glucose levels [30]. In light of the present findings, *KCNQ1* may have different effects on the complex lipid and glucose phenotypes.

There are some advantages and disadvantages in our study. The major advantage of the present study is the population-based approach, which overcomes the potential selection bias associated with hospital-based designs. However, this study also has some limitations: first, only four important SNPs in the *KCNQ1* gene were examined for possible association with metabolic-related parameters, although other associated variants may exist; second, no functional experiments were performed to validate our results; third, the sample size of the present study (N = 2963) was relatively small, which limited the statistic power for identifying associations between genetic variants and phenotypes with low prevalence rate (i.e., the fact that only 20 subjects had type 2 diabetes prohibited us to measure the relationship of these variants with diabetes risk).

In summary, we found that several polymorphisms in the *KCNQ1* gene were associated with metabolic phenotypes, especially TG levels, in Han Chinese and/or in Uyghur populations. Further studies are needed to replicate our findings in other East Asian populations.

## Materials and Methods

### Subjects

The Uyghur subjects in this study included 478 individuals (273 men and 205 women with a mean age of 52.95±11.28 years) randomly recruited from March to May 2005 and April 2006 from four villages in Tulupan District, Xinjiang Uyghur Autonomous Region, China. The 2,485 Han subjects in this study were randomly selected from the baseline samples of the Taizhou Longitudinal Study (TZL). TZL, which was initiated in July 2007, was an open-ended prospective study with very broad research aims. The design and baseline characteristics of this study have been previously described [31].

Subjects who reported receiving medications for the treatment of metabolic-related diseases were excluded. Subjects who reported having tumors, autoimmune diseases, or hematological diseases were also excluded. Written informed consent was obtained from each participant, and all protocols were approved by the Human Ethics Committee of Fudan University.

### Data collection

A standardized interview was conducted by trained personnel, and detailed information was collected concerning medical history and lifestyle characteristics, such as smoking and alcohol consumption. All participants received a physical examination and blood tests at a local hospital after overnight fasting. A standardized mercury sphygmomanometer was used to measure SBP and DBP, and these measurements were performed by two cardiologists. Body weight and height were measured with subjects wearing only light indoor clothing and without shoes. BMI was calculated by dividing weight (kg) by height squared (m^2^). The waist circumference was measured midway between the caudal point of the costal arch, as palpated laterally, and the iliac crest. The hip circumference was measured at the symphysis-trochanter femoris level. WHR was calculated by dividing the waist circumference by the hip circumference. The blood specimens were drawn after overnight fasting, immediately subjected to centrifugation, and analyzed within 8 h for Glu, TG, and TC. The distribution of the clinical parameters is listed in Table 5.

**Table 5:** Clinical characteristics of studied subjects.

	Uyghur	Han
Number of subjects	478	2,485
Male/Female	273/205	1253/1232
Smoking(yes/no)	104/372	104/856/1525
Alcohol drinking(yes/no)	30/439	765/1720
Tea drinking(yes/no)	N.M	647/1835
Age(year, mean ± SD)	52.95±11.28	57.12 ±12.16
BMI(kg/m^2^, mean ± SD)	25.57±4.21	23.91±3.53
WHR(mean ± SD)	0.88±0.09	0.89±0.06
SBP(mm Hg, mean ± SD)	134.25±26.70	126.89±18.67
DBP(mm Hg, mean ± SD)	81.61±14.62	83.59±10.58
LDL(mmol/L, mean ± SD)	N.M	1.80±1.03
HDL(mmol/L, mean ± SD)	N.M	1.37±0.19
Glu(mmol/L, mean ± SD)	5.57±1.49	5.88±1.71
TG(mmol/L, mean ± SD)	1.06±0.35	1.06±0.94
TC(mmol/L, mean ± SD)	4.24±1.02	4.36±1.75

Smoking status in Han indicated never/former/current.

N.M = no measurement.

### Genotyping

Blood samples were collected into EDTA-containing receptacles, and genomic DNA was extracted using a standard method. Sample DNA (10 ng) was amplified by PCR according to the manufacturer's instructions. Genotyping of the four SNPs in *KCNQ1*, rs1057128(G>A), rs2237892(C>T), rs2237895(A>C), and rs12720449(C>G) was performed using the TaqMan SNP genotyping assay (Applied Biosystems, Foster City, CA, USA). The genotyping success rates and concordance rates at all four loci were >99% based on 12% duplicate samples (n = 380).

### Statistical analysis

Deviation from Hardy–Weinberg expectation for the variants was tested using the chi-square statistic. Haplotype inference was conducted using Haploview software [32]. We treated the metabolic phenotypes (BMI, WHR, SBP, DBP, FBG, LDL, HDL, TG, and TC) as quantitative traits. Because these traits exhibited a skewed distribution, we normalized these traits using the Box-Cox method, which can be used to automatically identify a suitable power transformation for the data. To estimate the association of the variants with the metabolic parameters, we applied a general linear regression model under an additive model, adjusting for sex, age, smoking, and drinking, with the normalized BMI, WHR, SBP, DBP, FBG, LDL, HDL, TG, and TC. Each SNP was separately entered into the model as the explanatory variable. A pre-specified threshold of *p*<0.0125 was used for multiple correction significance (corresponding to *p*<0.05 after adjusting for four loci). We also performed a principal component analysis on the nine variables representing metabolic phenotypes in the Han population. The association of the variants in *KCNQ1* with changes in the extracted principal components was estimated using a general linear regression model. All analyses were performed using SAS statistical software (release 8.2, SAS Institute Inc, Cary, NC, USA).
